# Form of Petition to Legislature

**Published:** 1890-10

**Authors:** 


THE PETITION.

*To the Honorable the Senate and House of Representatives of the State of Texas*:

Your petitioners would respectfully represent that the absence of a public health system in our State leaves the citizens without any protection from epidemic or contagious disease; in view of this alarming fact we most urgently pray your honorable bodies that you will, without delay, frame such enactments as in your wisdom, will meet the necessity, by providing a general health system.

Furthermore, in consequence of there being no efficient law preventing the practice of medicine by incompetent and ignorant persons, we most earnestly and emphatically ask that such a law be made as will require all persons who may hereafter seek to engage in the practice of medicine to give evidence to a legally constituted authority, of their fitness and competence for the exercise of the responsible privilege, no matter how, when nor where *acquired;* in order that the people may be enabled to distinguish the true and qualified physician, from the mere pretenders to medical knowledge. Respectfully submitted.

